# Associations of Trunk Fat Depots with Insulin Resistance, β Cell Function and Glycaemia - A Multiple Technique Study

**DOI:** 10.1371/journal.pone.0075391

**Published:** 2013-10-08

**Authors:** Anjali Ganpule-Rao, Charudatta Joglekar, Deepak Patkar, Manoj Chinchwadkar, Dattatreya Bhat, Himangi Lubree, Sonali Rege, Bhagyashree Uradey, Chittaranjan Yajnik, John Yudkin

**Affiliations:** 1 Diabetes Unit, KEM Hospital, Rasta Peth, Pune, India; 2 Dr Balabhai Nanavati Hospital, Mumbai, India; 3 Department of Medicine, University College London, London, United Kingdom; University of Ulm, Germany

## Abstract

**Objective:**

Central (truncal) adiposity is associated strongly with insulin resistance and diabetes. There are very few reports comparing methods of trunk fat measurement in their ability to predict glycaemia and insulin resistance. We report a comparative analysis of different trunk fat measurements in predicting glycaemia and insulin resistance in middle aged Indian men.

**Materials and Methods:**

Trunk fat measurements were performed using anthropometry, magnetic resonance imaging (MRI), dual-energy X-ray absorptiometry (DXA) and computed tomography (CT) on 128 men. Additional measurements were taken to characterise insulin resistance (Matsuda index) and beta cell function (Insulinogenic Index), glycaemia (fasting and 120 min glucose concentrations). Using residual approach we compared the ability of different trunk fat measurement techniques to predict insulin resistance, beta cell function and glycaemia.

**Results:**

There was a strong association between trunk fat measures from each technique with glycaemia and insulin resistance indices but not with the Insulinogenic Index. Insulin resistance and glycaemia, were best predicted using anthropometric measurements, notably by waist circumference and subscapular skinfold thickness. Neither MRI measures of trunk or visceral fat nor DXA trunk fat added significantly. CT liver density contributed to some extent to predict insulin resistance and 120 min glucose after anthropometric measurements.

**Conclusions:**

Our results suggest that, in Indian men, anthropometric measurements are good predictors of glycaemia and insulin resistance. Other complex measurements such as MRI, DXA and CT make only a small addition to the prediction. This finding supports the application of anthropometry for determining trunk fat in clinical and epidemiological settings.

## Introduction

Obesity is a strong risk factor for type 2 diabetes because of its associations with insulin resistance. Central (truncal) obesity has been found to associate more strongly with insulin resistance and with diabetes than generalized obesity [1]–[3]. Epidemiological research generally uses anthropometric measures such as body mass index (BMI) and waist circumference (perhaps as a waist-to-hip ratio) and truncal skinfold thicknesses as measures of obesity as they are cheap to perform and universally available. But these do not accurately represent body fat and its distribution in specific regions. In the past two decades the development of body composition techniques such as dual-energy X-ray absorptiometry (DXA) has allowed quantification of ‘truncal’ fat, while techniques such as Magnetic Resonance Imaging (MRI) have made it possible to distinguish between its subcutaneous and intra-abdominal (visceral) compartments. It has also become clear that intra-hepatic fat plays an important role in insulin resistance [4]. To date, various studies have explored the association between fat distribution and insulin resistance/dysglycaemia using either anthropometry [2], or CT scan measures of subcutaneous and visceral fat, or [5], DXA [6], [7], or steatohepatosis [8]. But because these studies have used these techniques in isolation, these relationships remain poorly understood.

The CRISIS study has, for the first time measured total body fat, and truncal fat distribution by all 4 measures in the same 128 subjects, all men, of a limited range of age, and of single ethnicity. We have also employed a novel way of avoiding issues of collinearity of closely correlated variables. We measured body fat and its distribution by anthropometric techniques (waist circumference and skinfolds), as well as by MRI, DXA and CT, to assess the ability of these measurement techniques to predict glycaemia, insulin resistance and beta cell function. Our hypothesis was that the addition of precise measures of truncal fat by imaging techniques to anthropometric measures of truncal fat will improve the prediction of insulin resistance and glycaemia.

## Methods

### Ethics Statement

The study was approved by the Ethics Committee of the King Edward Memorial Hospital Research Centre. Informed consent was signed by all participants.

Details of the CRISIS study have been published previously. [9], [10] In short, the CRISIS study used multistage stratified random sampling to recruit 441 men between 30 and 50 years of age from in and around Pune (149 rural, 142 slum residents and 150 middle class residents). Anthropometry was performed on all recruits to the study. Those known to have diabetes, hypertension, or coronary heart disease during enrollment were excluded from the study. A random selection of 50 men from each of the tertiles of BMI distribution was chosen for study of body composition using DXA, MRI and CT and the association of these, and anthropometry, with metabolic risk factors. The study took place between April 2000 and June 2001.

Participants reported at the Research Centre the evening before the study and ate a standard dinner. After an overnight fast, an antecubital vein was cannulated and three fasting blood samples were drawn 5 min apart. Fasting values for glucose and insulin were determined as the mean of three samples. A 75 g oral glucose tolerance test (OGTT) was performed, with blood samples collected at 30 and 120 min.

### Trunk Fat Measurement

**Figure 1:**
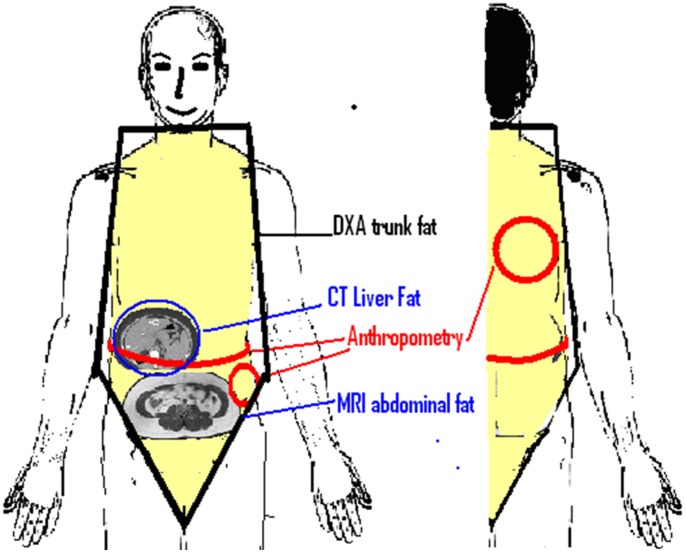
Trunk fat measurements by Anthropometry, MRI, DXA and CT.

Trunk fat was measured by four techniques (Figure 1).

Anthropometry – waist circumference, subscapular and suprailiac skinfolds

MR Imaging – subcutaneous and visceral fat from the xiphisternum to the pubic symphysis

DXA –trunk fat in dorsal and thoracic regions

CT- liver density (inverse relationship with liver fat content)

BMI and DXA total fat were also measured for the correlation matrix.

### Anthropometry

Trained researchers performed standardized anthropometric measurements, which included weight, height, waist and hip circumferences and skin fold thicknesses (subscapular and suprailiac). Two measurements were performed for each and the mean was used in the analysis. Inter-observer variation studies were conducted every three months and CV between observers was <2%.

### MR Imaging

MR imaging was performed with a 1 Tesla imaging device (Sigma Horizon LX, GE Medical Systems, San Francisco, CA). T1 weighted spin echo images were obtained axially through the abdomen from the xiphisternum to the pubic symphysis using the following imaging parameters: 10 mm slice thickness, 1 mm gap, 300 ms repetition time, 15 ms echo time and one-half excitation for all acquisitions. Images were acquired on a 256×192 matrix within a 48 cm×48 cm field of view and printed using automated imaging equipment (DV8100, Kodak, USA), scanned at 600 DPI resolution (Duotone 1200, Agfa, Germany) to convert into bitmap files. Each pixel on the scanned image represented 0.27 mm×0.27 mm of area of the abdomen (0.073 mm^2^). Each slice was processed by one of two trained radiologists by mapping the compartments midway between the anterior and posterior abdominal wall. Visceral and subcutaneous adipose tissue was measured using standard anatomical points [11]. All associations of abdominal fat were done employing volume data. Coefficient of inter-observer variation was calculated for 5 subjects (total of 148 sections and 6 compartments in each section). There was no statistically significant difference in the number of pixels counted in the same compartment by the two observers (mean difference: 438 pixels, 95% CI: −733 to 1610; P = 0.46). Inter-observer CV was 0.85%.

### Dual X-ray Absorptiometry (DXA)

Body composition was measured using a Lunar DPX-IQ 240 pencil beam machine (Lunar Corporation, Madison, WI, USA). Quality assurance tests were conducted every day following the manufacturer’s guidelines. Whole body scans were performed and were analysed using software version 4.7 [12] ^.^ The trunk fat region was defined using specific anatomic landmarks as advised in the manual (Figure 1). The coefficient of variation (CV) between 3 observers for regional measurements was <2%.

### Liver Density Measured by Computed Tomography (CT Liver Density)

The liver parenchymal density in Hounsfield units was measured on non-contrast CT (Siemens, ARC) scans using regions-of-interest in both the right and left hepatic lobes, avoiding the inclusion of portal or hepatic venous structures. Five measurements were taken for each and the mean was used in the analysis. CT liver density decreases in proportion to fat deposition, [8] so when CT liver density is used in the models, the relationships are in the reciprocal direction to those of liver fat.

### Laboratory Methods

Plasma glucose concentration was measured on a Hitachi 911 analyser (Hitachi, Tokyo, Japan) using the glucose oxidase method (intra- and inter-batch CV <4%). Insulin concentration was measured using in-house DELFIA method. [13] The UK National External Quality Assessment Service (UKNEQAS) (Guildford Peptides, Guildford, UK) results showed that the CV was 12.5% at <45 pmol/l, 9.6% at 45–90 pmol/l and 4.3% at >90 pmol/l.

### Terms, Calculations and Classification

Subjects were classified as per WHO BMI criteria into underweight (<18.5 kg/m^2^), normal (18.5 to 24.9 kg/m^2^), overweight (25.0 to 29.9 kg/m^2^) and obese (≥30.0 kg/m^2^). Glycaemic classification is shown by WHO (75 g OGTT) criteria [14]. Insulin resistance was calculated using the Matsuda index [15] and beta cell function by the Insulinogenic Index. [15] The Matsuda Index is derived from glucose and insulin concentrations during the OGTT and is believed to represent muscle as well as liver insulin sensitivity. The Insulinogenic Index, again derived from glucose and insulin concentrations during an OGTT, is a measure of beta-cell responsiveness to the oral glucose challenge.

### Statistical Methods

Data are presented as median (interquartile range, IQR) or as percent. Variables with skewed distribution (fasting glucose, 120 minute glucose, Matsuda index and Insulinogenic Index) were log transformed to satisfy normality. Primary analyses examined the associations of different trunk fat measurements with glycaemia, insulin resistance, and beta cell function. Pearson correlations between exposures (anthropometry, MRI, DXA, CT liver density) and outcomes (fasting glucose, 120 min glucose, Matsuda Index, and Insulinogenic Index) were calculated.

Secondary analyses used multiple regression to study the association between outcomes of interest and exposures. Age and place of residence were included in the model as potential confounders. Because there was substantial collinearity between different measures of trunk fat (r>0.5) these could not be used in a single model, so a residual approach was taken instead. Residuals were calculated by regression analysis of the two related exposures (trunk fat measures), thus giving the portion of second exposure independent of the first exposure. The residual thus calculated was used as the second exposure variable and so on. At each subsequent step only those exposures which were significant until the last step were included. We used this approach to generate exposure variables which were independently significant within the cluster of anthropometric (waist circumference, subscapular and suprailiac skinfold) and of MRI variables (visceral and subcutaneous fat). In the combined models we also included the anterior and posterior subcutaneous fat subdivisions of the MRI, when total subcutaneous fat mass made a significant contribution. For DXA trunk fat and CT liver density there is only one variable, so this process was not necessary, and the measurements were used as such in the final model. Thus, the final model for each outcome included relevant independent anthropometric and MRI variables along with DXA trunk fat and CT liver density. This process was repeated using different orders of independent variables to avoid the bias created by the order. All statistical analysis was performed using SPSS for Windows (version 16.0). The results of multiple linear regression analysis are shown using Standardized beta coefficients and change in r^2^ for every model.

## Results

The trunk fat measurements obtained by the different methods (anthropometry, MRI, DXA and CT) were available on 128 men (42 rural, 47 urban slum and 39 urban middle class residents). Details of the characteristics are shown in Table 1 for all subjects, and we have previously published detailed descriptions of the 3 separate groups [10]. The median height was 165.0 cm, weight 61.1 kg, and BMI 21.3 kg/m^2^. Of the 128 men studied, 23 (18%) were overweight and 3 (2%) were obese. Median total body fat by DXA, was 12.2 kg. Ten men (7.8%) had impaired fasting glucose, 17 (13.4%) had impaired glucose tolerance and 7 (6.2%) were diabetic by WHO criteria.

**Table 1:** Body composition and glycaemic measurements (n = 128).

Anthropometry	Median (25^th^,75^th^ centile)
Age (y)	39.0 (34.0, 45.0)
Height (cm)	165.0 (161.8, 169.4)
Weight (kg)	61.1 (54.3, 69.3)
BMI (kg/m^2^)	21.3 (19.5, 24.5)
Waist (cm)	84.1 (76.8, 91.8)
Subscapular skinfold (mm)	16.2 (11.3, 24.9)
Suprailiac skinfold (mm)	19.9 (11.8, 27.3)
**MRI**	
Total abdominal fat (kg)	4.84 (2.81, 7.12)
Subcutaneous fat (kg)	2.48 (1.37, 3.65)
Intraperitoneal fat (kg)	1.59 (1.00, 2.60)
**DXA**	
Total fat mass (kg)	12.22 (7.34, 17.79)
Trunk fat mass (kg)	6.50 (4.03, 10.18)
**CT**	
Liver density (Hounsfield Units)	66.0 (62.4, 68.4)
**Plasma glucose (mmol/L)**	
Fasting	5.1 (4.8, 5.6)
30 min	8.5 (7.4, 9.8)
120 min	6.2 (5.3, 7.2)
**Glucose tolerance**	
WHO (1999) (%)	
Normal	81
Impaired Glucose Tolerant	13
Impaired Fasting Glucose	8
Diabetic	6
**Plasma insulin (pmol/L)**	
Fasting	42.5 (27.1, 56.1)
30 min	361.1 (190.4, 596.7)
120 min	234.9(138.9, 504.0)
Insulin Sensitivity Index (Matsuda)	6.8 (4.5, 10.1)
Insulinogenic Index (β cell function)	5.8 (2.9, 9.1)

Table 2 shows the univariate correlation matrix of the anthropometric and metabolic variables. The different measures of trunk fat showed strong correlation with each other and to a weaker degree with liver density, adjusted for the known confounders of age and place of residence. All trunk fat measurements correlated inversely with Matsuda Index with correlation coefficients between 0.45 and 0.70, and more weakly with fasting (r = 0.14–0.25) and 120 min glucose (r = 0.24–0.44) concentrations. There were much weaker, and generally insignificant, correlations of trunk fat measurements with Insulinogenic Index. When these relationships were explored without adjustment for age and place of residence, the correlation coefficients were somewhat smaller (generally by 0.03–0.06).

**Table 2:** Pearson correlation coefficients within and between trunk fat exposures, glycaemia, insulin sensitivity and β-cell function.

Adjusted for age and place of residence	BMI	Waist	SS	SU	MRIs/c fat	MRI ants/c fat	MRI posts/c fat	MRI visc fat	DXA total fat	DXA trunk fat	CT liver density	Fglucose	120 min glucose	MATSUDA	Insu Index
BMI	**1**	**0.93**	**0.76**	**0.77**	**0.81**	**0.78**	**0.82**	**0.70**	**0.90**	**0.89**	−**0.47**	**0.27**	**0.32**	−**0.67**	**0.18**
Waist		**1**	**0.77**	**0.83**	**0.81**	**0.77**	**0.82**	**0.73**	**0.92**	**0.93**	−**0.46**	**0.30**	**0.33**	−**0.70**	**0.21**
SS			**1**	**0.74**	**0.64**	**0.60**	**0.65**	**0.60**	**0.76**	**0.78**	−**0.43**	**0.34**	**0.39**	−**0.58**	0.11
SU				**1**	**0.76**	**0.72**	**0.76**	**0.64**	**0.83**	**0.83**	−**0.41**	**0.31**	**0.34**	−**0.66**	**0.17**
MRI s/c fat					**1**	**0.98**	**0.99**	**0.78**	**0.87**	**0.86**	−**0.39**	0.17	**0.25**	−**0.51**	0.06
MRI ant s/c fat						**1**	**0.93**	**0.74**	**0.84**	**0.83**	−**0.39**	0.14	**0.24**	−**0.46**	0.02
MRI post s/c fat							**1**	**0.79**	**0.87**	**0.86**	−**0.37**	**0.18**	**0.26**	−**0.53**	0.09
MRI visc fat								**1**	**0.87**	**0.76**	−**0.38**	**0.25**	**0.35**	−**0.56**	0.08
DXA total fat									**1**	**0.99**	−**0.44**	**0.21**	**0.26**	−**0.64**	**0.20**
DXA trunk fat										**1**	−**0.47**	**0.25**	**0.31**	−**0.65**	**0.18**
CT liver density											**1**	−**0.18**	−**0.44**	**0.49**	−0.03
Fasting glucose												**1**	**0.68**	−**0.38**	−0.08
120 min glucose													**1**	−**0.49**	−**0.21**
MATSUDA														**1**	−**0.41**
Insu index															**1**

Key – SS: Subscapular skinfold, SU: Suprailiac skinfold, MRI: magnetic resonance imaging, DXA: dual-energy X-ray absorptiometry, CT: Computed tomography.

s/c: subcutaneous, MATSUDA: Matsuda Index, Insu index: Insulinogenic index.

The p values for all the correlations highlighted in bold are <0.01.

In order to explore the relationships of different measures of truncal fat to these metabolic variables, we constructed a series of multiple linear regression analysis (MLRA) models (Table 3 and 4). As these measures were closely interrelated, we used the residual approach as outlined in the statistical methods. First, we identified the dominant predictor of different outcomes for each method of trunk fat measurement (Anthropometry, MRI, DXA, CT) (Table 3). We then entered these predictors in a model for each outcome to get the best predictors. The results are described in detail for Matsuda Index and summarised for other outcomes.

**Table 3:** Modeling of trunk fat measurements to predict Matsuda Index (Individual Models).

		Exposures	Std β	% r^2^	p
		Age	0.086	0.1	0.31
		Place of residence	−0.394	15.0	**<0.0005**
		**Sequence 1**			
		Waist	−0.667	41.0	**<0.0005**
		R-subscapular	−0.060	0.5	0.32
		R-suprailiac	−0.097	0.1	0.108
	**Anthropometry**	**Sequence 2**			
		Waist	−0.667	41.0	**<0.0005**
		R-suprailiac	−0.110	0.8	0.068
**Trunk fat** **measures**		R-subscapular	−0.030	−0.2	0.621
		**Sequence 3**			
		Subscapular	−0.561	28.7	**<0.0005**
		R-suprailiac	−0.295	8.5	**<0.0005**
		R-waist	−0.215	4.4	**<0.0005**
		**Sequence 4**			
		Suprailiac	−0.631	35.9	**<0.0005**
		R-subscapular	−0.128	1.3	**0.044**
		R-waist	−0.288	4.4	**<0.0005**
		**Sequence 1**			
		Visceral	−0.541	26.1	**<0.0005**
	**MRI**	R-subcutaneous	−0.108	0.7	0.121
		**Sequence 2**			
		Subcutaneous	−0.515	21.6	**<0.0005**
		R-visceral	−0.236	5.2	**<0.0005**
	**DXA**	**DXA Trunk fat**	−0.652	35.5	**<0.0005**
	**CT**	**CT liver density**	0.481	19.1	**<0.0005**

R- Residual of the respective exposure.

**Table 4:** Combined Models for Matsuda Index.

	Exposures	Std β	% r^2^	p
	Age	0.086	0.1	0.31
	Place of residence	−0.394	15.0	**<0.0005**
Model-1	Waist	−0.667	41.0	**<0.0005**
	R-Visceral	−0.073	0.2	0.22
	R-DXA trunk fat	−0.027	−0.3	0.65
	R-CT liver density	0.150	2.0	**0.012**
Model-2	Visceral	−0.541	26.1	**<0.0005**
	R-Waist	−0.387	15.1	**<0.0005**
	R-DXA trunk fat	−0.027	−0.3	0.65
	R-CT liver density	0.150	2.0	**0.012**
Model-3	Waist	−0.667	41.0	**<0.0005**
	R-DXA trunk fat	−0.002	−0.4	0.978
	R-Visceral	−0.078	0.3	0.195
	R-CT liver density	0.150	2.0	**0.012**
Model-4	DXA trunk fat	−0.652	35.5	**<0.0005**
	R- Waist	−0.231	5.1	**<0.0005**
	R-Visceral	−0.078	0.3	0.195
	R-CT liver density	0.150	2.0	**0.012**
Model-5	Waist	−0.667	41.1	**<0.0005**
	R-CT liver density	0.152	1.9	**0.011**
Model-6	CT liver density	0.481	19.6	**<0.0005**
	R-Waist	−0.481	23.4	**<0.0005**

R- Residual of the respective exposure.

### Insulin Resistance (Matsuda Index)

As shown in table 3, we first entered age and place of residence as exposures in MLRA, followed by the first anthropometric measurement, waist circumference. Then we calculated the residual for the next anthropometric measure (subscapular skinfold thickness) using age, place of residence and waist circumference as exposures, and used this residual (R-) for the next step in the MLRA and so on. Using this approach we estimated the independent contribution of interrelated exposures. As there were multiple measures for anthropometry we tested all possible sequences (Table 3). Of anthropometric measurements, only waist circumference contributed to prediction of Matsuda Index (r^2^ = 41.0%) with no additional contribution from subscapular and suprailiac skinfolds (Sequences 1 and 2). Even when waist circumference was added to the model after skinfold thicknesses (Sequence 3 and 4), its contribution remained significant, implying that it was the dominant anthropometric predictor of Matsuda Index.

We used a similar approach to study independent associations with other trunk fat measurements (MRI, DXA trunk fat and CT). As seen in Table 3, visceral fat was the dominant MRI predictor of Matsuda Index (r^2^ = 26.1% ), and subcutaneous fat did not make a further significant contribution. DXA trunk fat explained 35.5% of the variance in Matsuda Index while CT liver density explained 19.1%, in both cases combined with age and place of residence. We then constructed a model by combining dominant predictors from each of the 4 measures of trunk fat (waist circumference; MRI visceral fat; DXA trunk fat; and CT liver density) (Table 4). After waist circumference, MRI-visceral fat and DXA trunk fat did not add significantly to the prediction of Matsuda Index, while CT liver density contributed a further 2%.

In an additional model, total fat measured by DXA contributed significantly (33.8% of partial r^2^, p<0.001) after age and place of residence (data not shown in table) but it did not contribute significantly after waist circumference and CT liver density measures to predict Matsuda Index.

### Insulinogenic Index and Glycemia

A similar approach was used to explore the relationship of trunk fat measures with Insulinogenic Index and glycemia (Table 5). Waist circumference and DXA trunk fat were significant predictors of Insulinogenic Index, with MRI or CT liver density not making further contribution over age and place of residence. Subscapular skinfold thickness was the dominant anthropometric determinant of both fasting and 120 min plasma glucose concentration. Of MRI, visceral fat was the dominant predictor, while for the other trunk fat techniques; DXA trunk fat and CT liver density were significant predictors of fasting and 120 min plasma glucose concentration.

**Table 5:** Modeling of trunk fat measurements to predict Insulinogenic Index and glycemia (Individual Models).

		Insulinogenic index			Fasting glucose			120 min glucose		
	Exposures	Std β	% r^2^	p	Std β	% r^2^	p	Std β	% r^2^	p
	Age	−0.163	1.8	0.07	0.084	0.1	0.348	0.072	−0.3	0.409
	Place of residence	0.132	1.0	0.143	0.248	5.3	**0.005**	0.323	9.5	**<0.0005**
**Anthropometry**	**Sequence 1**									
	Waist	0.230	4.0	**0.024**	0.301	7.6	**0.001**	0.324	9.4	**<0.0005**
	R-subscapular	−0.094	0.2	0.282	0.168	2.2	**0.043**	0.200	3.5	**0.013**
	R-suprailiac	0.004	−0.8	0.96	0.064	−0.3	0.438	0.064	−0.3	0.419
	**Sequence 2**									
	Waist	0.230	4.0	**0.024**	0.301	7.6	**<0.0005**	0.324	9.4	**<0.0005**
	R-suprailiac	−0.027	−0.7	0.757	0.110	0.5	0.187	0.114	0.7	0.160
	R-subscapular	−0.091	0.1	0.304	**0.142**	−0.6	0.087	0.177	2.5	**0.027**
	**Sequence 3**									
	Subscapular	0.115	0.4	0.22	0.343	10.2	**<0.0005**	0.385	13.4	**<0.0005**
	R-suprailiac	0.132	1.0	0.14	0.083	0.0	0.314	0.073	−0.1	0.354
	R-waist	0.165	2.0	0.063	0.03	−0.7	0.973	−0.011	−0.7	0.886
	**Sequence 4**									
	Suprailiac	0.179	2.1	0.058	0.313	8.4	**<0.0005**	0.337	10.2	**<0.0005**
	R-subscapular	−0.031	−0.7	0.732	0.157	1.8	0.058	0.190	3.1	**0.017**
	R-waist	0.165	2.0	0.063	0.003	−0.7	0.973	−0.011	−0.7	0.886
**MRI**	**Sequence 1**									
	Visceral	0.082	−0.2	0.388	0.259	5.4	**0.004**	0.348	12.7	**<0.0005**
	R-subcutaneous	−0.004	−0.8	0.967	−0.053	−0.3	0.547	−0.025	0.0	0.753
	**Sequence 2**									
	Subcutaneous	0.065	−0.5	0.515	0.177	1.8	0.065	0.266	5.6	**0.004**
	R-visceral	0.051	0.7	0.57	0.199	3.3	**0.024**	0.227	4.5	**0.006**
**DXA**	DXA Trunk fat	0.193	2.3	**0.05**	0.268	5.3	**0.005**	0.324	8.5	**<0.0005**
**CT**	CT liver density	−0.031	−0.8	0.746	−0.187	2.3	**0.043**	−0.439	16.7	**<0.0005**

R- Residual of the respective exposure.

In the combined model (Table 6) that included dominant predictors from each of the trunk fat measurements waist circumference was the significant predictor for Insulinogenic Index. For fasting glucose subscapular skinfold thickness alone, while for 120 min glucose both subscapular skinfold thickness and CT liver density were dominant predictors.

**Table 6:** Combined Models for Insulinogenic Index and Glycaemia.

	Insulinogenic Index			Fasting glucose			120 min glucose		
Exposures	Std β	% r^2^	P	Std β	% r^2^	p	Std β	% r^2^	p
Age,	−0.163	1.8	0.07	0.084	0.1	0.348	0.072	−0.3	0.409
Place of residence	0.132	1.0	0.143	0.248	5.3	**<0.005**	0.323	9.5	**<0.0005**
Waist	0.230	4.0	**<0.024**	–	–	–	–	–	–
Subscapular	–	–	–	0.343	10.2	**<0.0005**	.385	13.4	**<0.0005**
R-CT liver density	–	–	–	−0.037	−0.6	0.654	−0.277	7.3	**<0.0005**

R- Residual of the respective exposure.

Finally, we investigated whether the relationship of trunk fat measures with glycaemia is mediated by the effect of trunk fat on insulin resistance and beta-cell function (Table S1). In this analysis, after age and place of residence, we added Matsuda Index followed by the residual of insulinogenic index, and finally the residuals of the dominant trunk fat measures. In the model that included Mastuda Index, trunk fat measurements did not make a significant contribution to the variance of glycaemia. Overall for insulin resistance, beta cell function measures and glycaemia, anthropometric measurements were the dominant predictors.

## Discussion

The association of central obesity with insulin resistance is well recognized. In our study we have been able to explore the relative contributions of different central fat depots, measured using a range of standard imaging techniques, with population measures of insulin resistance and of beta cell function. To the best of our knowledge, this is the first report on the association of simple to complex trunk fat measurements with glycaemia and its determinants in a group of adult Asian-Indian men. The present study suggests that anthropometric measurements are good predictors of glycaemia and one of its determinants – insulin resistance. Other complex measurements such as MRI, DXA and CT make only a small addition to the prediction after anthropometric measures. We adjusted the results for age and place of residence as possible confounders though the results were similar even without adjustment.

Central obesity is usually assessed by the easily measured waist circumference or waist-to-hip ratio, while use of sophisticated imaging techniques, such as DXA, MRI and CT allow accurate assessment of different fat depots. Similar to other studies our results show that anthropometric measurements, mainly waist circumference [16], [17] and DXA trunk fat [6], [18] can be used as a measure of central adiposity and for prediction of glycaemic risk and insulin resistance.

Our finding that visceral fat has stronger associations than subcutaneous fat with glycaemia and insulin resistance are compatible with findings from some [19], [20], but not all, prior research [5], [21], [22]. In these associations the dominant paradigm, the ‘Portal Hypothesis,’ implicates the higher rates of lipolysis of visceral fat, as well as the delivery of products of lipolysis, or pro-inflammatory cytokines, directly into the portal vein and the liver. Over the last decade, a series of observations from clinical physiology or animal models has led to challenges to this mechanism. Thus Misra [5] and Abate [21] have shown stronger correlations of truncal subcutaneous fat mass, particularly posterior, to insulin resistance than of visceral fat mass. The same group has also reported that the insulin resistance of south Asian subjects is unrelated to differences in intraperitoneal fat mass [23].

Lee and colleagues have presented a series of alternative hypotheses outlining ways in which central fat distribution might be related to insulin resistance, glucose intolerance and vascular disease [4], and have reviewed studies that challenge the tenets of the portal hypothesis. This series of clinical, animal and genetic studies raise questions about the link between visceral obesity, non-esterified fatty acids (NEFA) and hepatic insulin resistance and show that adipose signaling of insulin resistance is not mediated solely through NEFA. There is a growing recognition both that other products of adipose tissue, generated by both adipocytes and inflammatory cells, may play an important role in determining insulin resistance, and that the relative importance of NEFA and adipokines may differ between liver and skeletal muscle.

There is also a growing recognition that adipose tissue accumulation *per se* may not be sufficient to produce the low-grade inflammatory state of insulin resistance, and the hypothesis has emerged that ectopic fat may develop only when physiological storage capacity is exceeded [24], [25]. The tendency of Indian subjects to central obesity, insulin resistance and diabetes [2], [26] may be a consequence of a lower storage capacity. This possibility may mean that our observations are specific to Indian men and need repeating in women and in other ethnic groups.

There are very few studies that have reported associations of liver density using CT scanning with insulin resistance and glycaemia. In our study, CT liver density was strongly correlated with other measures of trunk fat and was a weak independent predictor of insulin resistance and 120 min glucose concentration. Dwyer studied changes in CT liver density with respect to increase in liver glycogen content [8], Banerji [20] has shown that liver fat content measured by CT is associated with visceral fat mass and serum triglyceride levels and inversely with glucose disposal, indicating that hepatosteatosis may contribute to insulin resistance and be a link between visceral adipose tissue mass and serum triglyceride levels.

Another important finding of our study is that associations between trunk fat measurements and glycaemia appear to be mediated in full by insulin resistance expressed as Matsuda. Matsudas both hepatic and peripheral insulin resistance [27] and its relationships to measures of posterior truncal fat and liver density parallel those of measures of glycaemia.

Various studies have compared trunk fat measured by different methods and have shown that all these methods are highly correlated. [7], [28]–[30] Studies comparing different methods of measurement of abdominal fat to predict insulin resistance [31] or glycaemia [32] have employed multivariate regression analyses to find the best predictors, but have never taken into account the strong co-linearity between the various fat compartments. We have employed an innovative residual approach to take account of this. We have also avoided first variable selection bias by analysing different permutations and sequences of the measures of trunk fat.

Ours is the largest study of trunk fat measurements by multiple methods, it is community based and we studied an ethnically homogenous population of the same gender within a narrow age band. Thus our study has larger power than previous studies to define abdominal fat distribution and its associations with metabolic variables. Measurement of both insulin resistance and beta cell function allowed us to construct a more complete picture of the pathogenesis of hyperglycaemia. The limitations of our study include the fact that we did not study women or subjects of other ethnicities, and we estimated insulin resistance by Matsuda Index, widely-accepted approaches to studying insulin resistance in large numbers, where clamp methods are inapplicable. The participants were relatively young men and 80% were of normal weight and 80% had normal glucose tolerance. The study may have shown different results in individuals with greater degrees of glucose intolerance and insulin resistance. Finally, ours is a cross-sectional study and therefore we cannot assert causality.

In conclusion, our results confirm importance of the anthropometric measurements as a surrogate marker of the abdominal fat in normal subjects. We also report that of the more sophisticated measures, only CT liver density adds to the prediction of insulin resistance after anthropometric measures, making a very small contribution to explained variance. While these observations are of limited application to clinical scenarios, it suggests that sophisticated research on fat distribution can be undertaken with low technology methods.

## Supporting Information

Table S1 **Combined Models for Insulinogenic Index and Glycaemia.**
(DOCX)
