# Supplementary material for: Associations of Trunk Fat Depots with Insulin Resistance, β Cell Function and Glycaemia - A Multiple Technique Study
Source: PLoS One. 2013 Oct 8;8(10):e75391. doi: 10.1371/journal.pone.0075391 (PMC3792935; doi:10.1371/journal.pone.0075391)
Supplement: Table S1 — Combined Models for Insulinogenic Index and Glycaemia. (DOCX) [file pone.0075391.s001.docx]

**Supplemental table-1**

**Effect of including Matsuda Index in models for glycaemia**

|  | Fasting glucose | | | 120min glucose | | |
| --- | --- | --- | --- | --- | --- | --- |
|  | Std β | % r^2^ | P | Std β | % r^2^ | p |
| **Matsuda Index** |  |  |  |  |  |  |
| Age | 0.084 | 0.1 | 0.348 | 0.072 | -0.3 | 0.409 |
| Place of residence | 0.248 | 5.3 | **0.005** | **0.323** | **9.5** | **<0.0005** |
| Matsuda | -0.408 | 13.0 | **<0.0005** | **-0.509** | **21.8** | **<0.0005** |
| R-Insulinogenic Index | -0.326 | 15.6 | **<0.0005** | **-0.446** | **23.4** | **<0.0005** |
| R-subscapular skinfold | 0.001 | -0.6 | 0.998 | -0.015 | -0.4 | 0.806 |
| R-visceral fat | - | - | - | 0.010 | -0.4 | 0.868 |
| R-CT liver density | - | - | - | -0.116 | 1.0 | 0.061 |

R- Residual of the respective exposure
